# Penfluridol as a Candidate of Drug Repurposing for Anticancer Agent

**DOI:** 10.3390/molecules24203659

**Published:** 2019-10-11

**Authors:** Nguyen Minh Tuan, Chang Hoon Lee

**Affiliations:** College of Pharmacy, Dongguk University, Seoul 04620, Korea; tuank67a5@gmail.com

**Keywords:** penfluridol, antipsychotic, drug repositioning, hallmarks of cancer, autophagy, glioblastoma

## Abstract

Penfluridol has robust antipsychotic efficacy and is a first-generation diphenylbutylpiperidine. Its effects last for several days after a single oral dose and it can be administered once a week to provide better compliance and symptom control. Recently; strong antitumour effects for penfluridol were discovered in various cancer cell lines; such as breast; pancreatic; glioblastoma; and lung cancer cells via several distinct mechanisms. Therefore; penfluridol has drawn much attention as a potentially novel anti-tumour agent. In addition; the anti-cancer effects of penfluridol have been demonstrated in vivo: results showed slight changes in the volume and weight of organs at doses tested in animals. This paper outlines the potential for penfluridol to be developed as a next-generation anticancer drug.

## 1. Introduction

According to the World Health Organization, the burden from cancer in 2018 was around 18.1 million new cases and 9.6 million cancer fatalities in 2018 [1]. Several cancers such as glioblastoma (GBM), metastasis of breast cancer, and pancreatic are a leading cause of mortality. GBM is an incurable brain tumour with a 12% survival rate. Based on statistics from several institutions, breast cancer is the most diagnosed cancer and ranked second among leading causes of death. Pancreatic cancer is ranked fourth among leading cause of cancer-related death. The onset of pancreatic cancer is mostly undetected, and patients with this condition are typically diagnosed at late stages, when the disease is almost resistant to chemotherapies. 

According to the US National Cancer Institute, cancer patients may have to pay more than $10,000 a month for individual drugs. Several anticancer drugs have witnessed a dramatic increase in price year-on-year after launch. In an eight-year appraisal of 24 injectable cancer-drug price in the United States, it was concluded that anticancer drugs tend to increase, regardless of competition in the anticancer drug markets [2]. In another study assessing price trends for 58 anticancer drugs approved in 1995 to 2003, price increases from launch, after adjusting for inflation and health benefits, were 10% per year (i.e., $8500 annually) [3]. Moreover, various treatments, ranging from surgery to systematic treatments, are needed for a cancer patient, ranging from surgery to systematic treatment. Hence, financial burden is a perennial problem for cancer patients that is gradually exacerbated. Drug repositioning may be a major way of overcoming the ‘economic toxicity’ of anticancer therapeutics. In searching for novel anticancer activity by drug repurposing, penfluridol was identified as a potent agent against malignant tumours. 

## 2. What is the Penfluridol?

Penfluridol, discovered in 1968 at Janssen Pharmaceutical, is an established first-generation antipsychotic drugs used in the treatment of chronic schizophrenia and other psychotic disorders. It has a long elimination half-life up to one week [4]. Clinical research suggested that the appropriate dosage for penfluridol was 40 to 80 mg/week [5].

**Figure 1:**
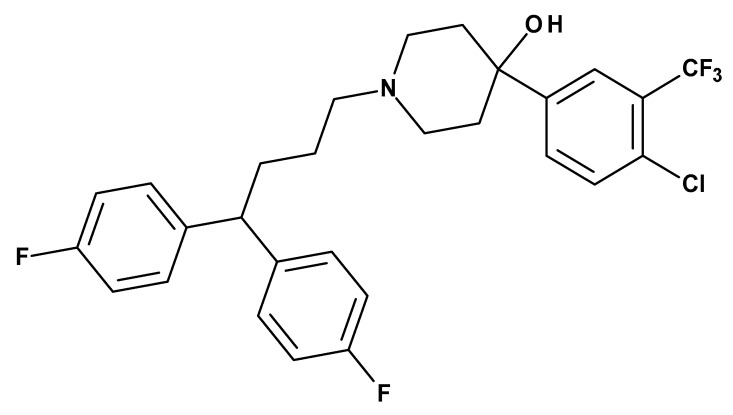
The chemical structure of penfluridol and some antipsychotic drugs.

The mechanism of penfluridol action against the positive symptoms of schizophrenia and other psychotic disorders is thought to be blockade of dopamine receptors, especially to postsynaptic D_2_ receptor [6,7] (Figure 1). Penfluridol also acts as a T-type calcium- channel blocker (Kd value ≈ 70–100 nM) [8]. 

Recently, measured binding affinity values, Ki (nM), for central nervous system G protein coupled receptors (GPCRs) and transporters related to the efficacy and side effects of penfluridol were 356 (5-hydroxytryptamine [serotonin] receptor 1A; 5HT_1A_), 3560 (5HT_1D_), 361 (5HT_2A_), 184 (5HT_2B_), 881 (5HT_2C_), 10,000 (5HT_5A_), 10,000 (5HT_6_), 280 (5HT_7_), 147 (dopamine receptor D1), 159 (D_2_), 136 (D_3_), 10,000 (D_4_), 125 (D_5_), 10,000 (κ-opioid receptor), 867 (μ-opioid receptor), 1714 (δ-opioid receptor), 10,000 (histamine receptor 1 H_1_), 10,000 (H_2_), 588 (norepinephrine transporter), 10,000 (serotonin transporter), 1714 (dopamine transporter), 602 (α_1D_-adrenoreceptor), 401 (α_2B_), 455 (α_2C_), and 515 nM (β_3_) [9]. These values were reported by Ashraf-Uz-Zamanem et al. [9] and supported by the National Institute of Mental Health’s Psychoactive Drug Screening Program. In particular, the Ki values of 184 (5HT_2B_), 147 (D_1_), 159 (D_2_), 136 (D_3_), and 125 nM (D_5_) seem to be more significant.

## 3. Anti-Cancer Effects of Penfluridol

Penfluridol has anticancer effects against various cancer cell lines via several underlying mechanisms (Table 1). For instance, in an orthotopic model of breast cancer, penfluridol suppressed breast cancer growth by 49% [10]. More compellingly, penfluridol suppressed the growth of metastatic brain tumours, after breast cancer cells were introduced by intracardiac and intracranial injection, by 90% and 72%, respectively [10]. Furthermore, penfluridol inhibited the growth of GBM cell lines, such as U87MG, after subcutaneous and intracranial in vivo GBM tumour models, by 65% and 72%, respectively [11]. Penfluridol also inhibited the growth of Panc-1, BxPC-3, and AsPC-1 pancreatic cancer cells with concentrations needed for 50% inhibition (IC_50_) of 6–7 μM after 24 h treatment [12].

**Figure 2:**
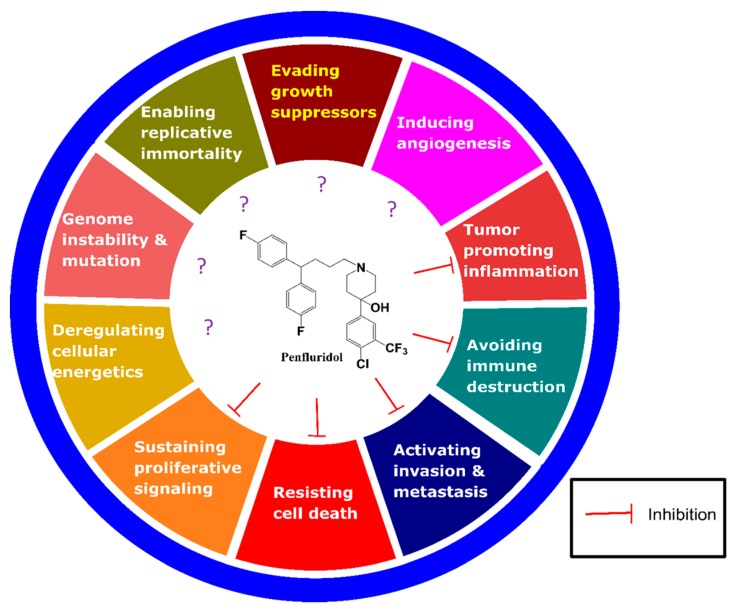
The hallmark of cancer. Inhibition mark (ㅏ) indicates the part inhibited by penfluridol and question mark (?) indicates the part not yet studied. Modified from Hanahan & Weinberg’s report [14].

We have described the anticancer activity of penfluridol in terms of the 10 hallmark aspects of cancer [14] (Figure 2).

### 3.1. Penfluridol Suppresses Cell Proliferation

**Figure 3:**
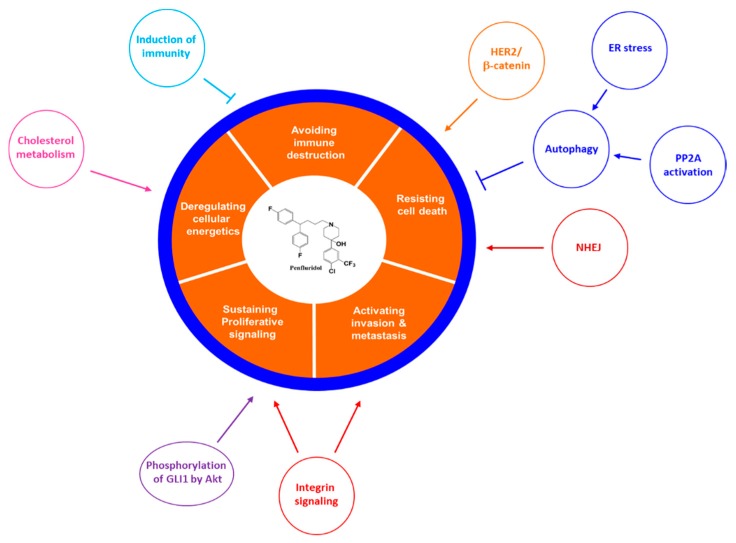
Inhibitory mechanisms of penfluridol on the hallmarks of cancer. Cancer hallmarks not yet studied with penfluridol are not shown in the figure.

Cell proliferation plays a pivotal role in tumourigenesis (Figure 2). In research performed in 2015, penfluridol suppressed pancreatic cancer cell proliferation in various cell lines, such as Panc-1, BxPc 3, and SU8686, at 12.0, 9.3, and 16.2 µM, respectively. The postulated mechanism was via protein phosphatase 2A (PP2A) [15], which is thought to be crucially involved in controlling cellular growth and has proved to be a potent anti-tumour target [16]. Subsequent studies supported these findings of antiproliferative activity via the autophagy [12]. In another study, cyclin D and Myc, which are associated with cell growth, were inhibited by penfluridol [15] (Figure 3).

### 3.2. Penfluridol Induces Cell Death

Apoptosis is implicated in the suppression of tumourigenesis but is often impaired in cancer [17] (Figure 2). Recently, penfluridol promoted apoptosis and autophagy, notably the apoptosis of metastatic breast cancer, even relative to paclitaxel (an established first-line treatment for metastatic cancer) in paclitaxel-resistant patients. Interestingly, penfluridol also reversed the resistance of breast cancer cells to paclitaxel [18]. Penfluridol had IC_50_ values of approximately 2 to 3 µM and 4 to 5 µM against cell lines sensitive to or resistant to paclitaxel, respectively [18]. 

Moreover, penfluridol induced apoptosis in GBM cells and pancreatic tumours [11,12]. Cyclin B1 and p21, which act as biomarkers of G-2 cell-cycle arrest, indicated suppression of MiaPaCa2 cells treated with 10 µM after 24h [15]. 

### 3.3. Penfluridol Impedes Metastasis and Invasion

Malignant tumours of one organ tend to invade surrounding tissues and spread to other organs; this process is called metastasis. Such invasion is one of the prominent characteristics of aggressive tumours and is also the biggest cause of cancer death [19] (Figure 2).

In a recent study, penfluridol proved to be a potential solution to in vivo metastasis of breast cancer to the brain. In vitro, a wound-healing assay revealed that penfluridol 4 µM inhibited 4T1 cell migration by up to 61% and 76% at 18 and 36 h, respectively [11]. This result was also supported by a Transwell-invasion assay, in which 60% of the cells migrated compared to the controls. In vivo, in a model of triple-negative breast cancer (TNBC), penfluridol had 90% and 72% anti-metastatic activity after intracardiac or intracranial injection, respectively, of 4T1 cancer cells [10].

### 3.4. Penfluridol Hinders Angiogenesis

Tumour secretes factors for angiogenesis for rapid growth, and new blood vessels form to support tumour growth [20] (Figure 2). To the best of our knowledge, no previous research exists about a direct relationship between penfluridol and suppression of angiogenesis; pimozide—a penfluridol derivative—has shown suppression of angiogenesis, potentially via inhibition of the Akt-signalling pathway [21]. CD31 level, a biomarker of angiogenesis [22,23]; was reduced by 78% in pimozide-treated mice compared with controls, and VEGFR1 and VEGFR2 levels (also biomarkers of angiogenesis) recorded in relative RNA expression of fibroblast cells were 4.33-fold and 1.66-fold lower than in controls, respectively [21]. These findings suggest that penfluridol may possess anti-angiogenic effect such as pimozide.

### 3.5. Penfluridol and Evading Immune Destruction

Regulatory T cells (Treg) have an important role in immunosuppression of tumour microenvironment [24] (Figure 2). Myeloid-derived suppressor cells (MDSCs) saturating malignant GBM may stimulate Tregs [25]. Tregs may also suppress M1 macrophages, which could kill malignant cells [26].

In 2007, penfluridol was shown to suppress Tregs that highly expressed FoxP3 and CD4^+^. In addition, M1 Macrophage (which produce CD86 and interleukin-12) increased after treatment with penfluridol. Thus, it is likely that penfluridol has highly potential to hinder the ability of cancer cells to avoid immune destruction [27].

### 3.6. Penfluridol and Inflammation

Inflammation is a feature of cancer that contributes significantly to tumour progression (Figure 2). Recent advances in resolution of inflammation suggest new perspectives about the role of inflammation in cancer [28].

One recent study evaluated lysis of U87MG tumours derived from nonobese diabetic/severe combined immunodeficiency mice injected with human peripheral blood mononuclear cells. Interferon-γ and C-C motif chemokine ligand 4, two biomarkers of inflammation with key roles in tumour progression, showed decrease expression in penfluridol-treated groups compared to control groups [27].

### 3.7. Penfluridol and Replicative Immortality

The population of most normal cells undergoes a finite number of doublings. Conversely, tumour cells require unlimited propagation for malignant growth. Cell proliferation without limitation in numbers is known as the replicative immortality [14] (Figure 2). There is no report that penfluridol directly inhibits replicative immortality. However, there is a report that voltage-gated calcium and L-type voltage-gated calcium channels are related to telomerase activity for unlimited replicative potential, which indicates that penfluridol may be involved in replicative immortality [29].

### 3.8. Penfluridol Increases the Efficiency of Growth Suppressors

Cancer cells tend to avoid growth suppressors associated with the downregulation of cell proliferation (Figure 2). Several tumour suppressors such as phosphatase and tensin homologue (PTEN), retinoblastoma protein (RB), and Tp53 may exert bona fide suppressive effects on cancer growth in various ways [14,30]. It has been reported that penfluridol activates PP2A, a growth suppressor, to inhibit the growth of pancreatic cancer [15].

### 3.9. Penfluridol and Genome Instability and Mutation

Normal cells transform into cancerous ones after the accumulation of gene mutations associated with cell growth suppressors and cell division [31]; genome instability and mutations are characteristic of most cancers [14] (Figure 2). There have been no reports about the use of penfluridol in relation to genome instability and mutation-related machinery. However, penfluridol has shown therapeutic efficacy in adult GBM patients with *IDH1* mutations [11].

### 3.10. Penfluridol and Deregulating Cellular Energetics

Reprogramming of energy metabolism is a characteristics of cancer [14] (Figure 2). Cancer cells appear to decrease effectiveness of ATP production, but it increases glucose uptake via compensatory upregulation of glucose transporters [14] (Figure 2). Penfluridol has not been associated with Warburg effects; however, it has shown anticancer activity via dysregulation of cholesterol homeostasis [13]. ATP deprivation mediated by penfluridol-induced accumulation of autophagosomes results in nonapoptotic cell death through unfolded protein response in lung cancer cell lines [32].

## 4. Mechanism of Action of Penfluridol on Cancer

### 4.1. The Antipsychotic-Related Mechanism of Action of Penfluridol on Cancer

#### 4.1.1. The Relationship between Dopamine Receptor D2 and Cancer

From 2003 onwards, several screening studies were conducted to evaluate D_2_-receptor antagonists as potential agents for cancer treatment, given that D_2_-receptors are present in various cancer cell lines. D_2_-receptor antagonists showed biological effects against cancer in vitro and in vivo [33]. Moreover, D_2_-receptor agonists increased phosphorylation at threonine 308 of Akt in neurons [34], and Akt phosphorylation is known to plays a vital role in cell proliferation; this suggests that D_2_- receptors are associated with tumourigenesis. Unlike D_2_- agonist, D_2_-antagonists decreased cell viability and encouraged apoptosis in several cancer cell lines in vitro [10,12,34,35,36,37,38].

The molecular mechanisms of D_2_-receptor antagonists against cancer cell growth have been recorded in attractive therapeutic targets such as signal transducer and activator of transcription, receptor tyrosine kinase, Wnt, phosphoinositide 3-kinase, and mitogen-activated protein kinase/extracellular signal-regulated kinase. Recently, D_2_-receptor antagonists mitigated cell proliferation and induce apoptosis in vitro in various cancer cell lines. In addition, D_2_-receptor antagonists had potent effects in some cancer xenograft animal models, suggesting that D_2_-receptor antagonist may be used as a chemotherapeutic target [33]. However, there is no direct evidence that the anticancer activity of penfluridol is due to D_2_-receptor antagonism. It is difficult to find a report explaining various mechanisms of anticancer activity involving of D_2_-receptor antagonism. In addition, penfluridol derivatives showed distinct anticancer and antipsychotic activities, thus suggesting that D_2_-receptor antagonism may or may not contribute to the anticancer activity of penfluridol [9]. 

#### 4.1.2. The Relationship between T-type Calcium Channels and Cancer

Calcium is an important second messenger with a pivotal role in cellular processes associated with cell proliferation, growth, and differentiation [39]. Overexpression of T-type calcium channels was recorded in many cancer cell lines compared with normal cells [40,41]. In addition, T-type calcium channels prevail in various cells in the body [42,43], and these channels may be involved in controlling the entry of extracellular calcium into the cells, which is important for cell-cycle progression [39,44]. Thus, T-type calcium channels were implicated in calcium-dependent biological processes associated with cellular growth, proliferation, and survival and may therefore be an effective anti-cancer target.

There are no reports that penfluridol directly inhibits cancer via the calcium channel. However, calcium channel blockade inhibits prolactin gene expression [45], inhibition of prolactin may be important in the treatment of advanced prostate cancer [46]. Therefore, via calcium channel inhibition, penfluridol may have a role in the treatment of advanced prostate cancer.

The mechanism of apoptosis induction related to penfluridol was studied in GBM. Results indicated that mTORC2/Akt axis induced apoptosis through blockade of T-type calcium channel [47].

### 4.2. The Novel Molecular Mechanism of Action of Penfluridol on Cancer 

Many mechanisms of actions are reported on the effects of penfluridol on the inhibition of cancer hallmarks (Figure 3).

#### 4.2.1. Inhibition of Integrin Signalling Pathway

When breast cancer metastasizes—especially to the brain—it is typically a death sentence for patients. Most anticancer agents cannot cross the blood-brain barrier (BBB), which poses a difficult challenge for effective treatment. Recently, penfluridol demonstrated antiproliferative activity against TNBC cell lines and against breast cancer metastasis to brain. These effects have been attributed, in part, to the molecular mechanism of penfluridol-induced suppression of integrin α6 and integrin ß4 in breast cancer cell lines and GBM [10,48].

To date, no relationship has been reported between D_2_ receptors, T-type calcium channels, and integrin pathways, although dopamine interactions with D_2_ and D_3_ receptor may induce integrin ß1 in normal human T cells [49]. Importantly, integrin expression plays a critical role in the anchorage of epithelial cells; without such adhesion, cells could not proliferate in response to growth factors. Integrin dysregulation may drive the formation of breast cancer, although integrins are not thought be *bona fide* oncogenes. Compelling evidence about the bridge between integrins expression and metastasis was found recently: research showed that when αvß3 integrin was activated, it could cause adherence of breast cancer to platelets, which would make disseminated cancer cells stay in the circulation before extravasation [50]. In addition, such integrins have a more direct impact on tumour cells by inducing intracellular signals that encourage tumour progression [51].

Other recent research showed that the suppressive effect of penfluridol on breast cancer cell lines, via inhibition of the α6β4 integrin, plays a crucial role in breast tumour progression. Penfluridol inhibited activation and expression of integrin downstream signalling mediated by focal adhesion kinase, paxillin, Rac, and Rho-associated protein kinase in vitro. Administration of penfluridol 10 mg/kg per day by oral gavage showed a strong suppressive effect against metastasis and growth of 4T1-luc cells in brains and fat pads. Mice showed no significant behavioural side effects, such as clockwise or counter-clockwise revolution, total distance moved, and horizontal and vertical activity measured by Versamax (Accuscan Instruments, Columbus, OH). In addition, mice showed no signs of toxicity, such as changes in bodyweight, plasma aspartate and alanine transaminase levels, and weights of kidney, brains, liver, and spleen, when treated with penfluridol, 10 mg/kg by oral gavage for 55 days. However, no conclusions about the neurological side effects of penfluridol can be drawn from this study [10,52]. Anyway, these results may suggest marked potential for penfluridol in the treatment of TNBC, which is currently considered untreatable [10].

In addition, integrin suppression was found in penfluridol-treated cells via induction of reactive oxygen species and downregulation of Sp transcription factors [53]. Tumourigenesis was attributed to these biological molecules [54,55]. Notably, Sp transcription factors were recognized as a target for anticancer agents [56].

In GBM, penfluridol suppressed cancer cell migration and invasion by reducing the expression of integrin α6 and uPAR and suppressing the expression of epithelial-to-mesenchymal transition (EMT) factors, vimentin and Zeb1 [48].

#### 4.2.2. Inhibition of Akt-Mediated Phosphorylation of Glioma-Associated Oncogene 1 (GLI1)

Glioma-associated oncogene 1 (GLI1) is a member of the sonic hedgehog pathway, which is overexpressed in GBM cancer cells [57]. In addition, the resistance of GBM tumours to current remedies is related to the extent of GLI1 overexpression [58]. Downregulation of Akt signalling was documented as T-type calcium channels, which were inhibited by a small-interfering (si)RNA-mediated knockdown. This resulted in the promotion of apoptosis in GBM cells and initially indicated a transparent connection between Akt signalling pathway and T-type calcium channels [45]. In another study, D_2_- receptors were implicated in regulation of Akt signalling [59]. These results suggested that penfluridol’s targets T-type calcium channel and D_2_ receptor might be implicated in the inhibition of Akt. However, it is not clear whether penfluridol directly inhibits Akt.

Penfluridol downregulated Akt phosphorylation at serine 473, octamer-binding transcription factor 4 (OCT4), Nanog, and Sox2, as well as the GLI1. Besides, GLI1 expression decreased in GBM cells treated with a PI3K/Akt inhibitor (LY294002) or Akt knocked down by Akt siRNA. Thus, penfluridol suppresses the proliferation and growth of GBM cancer cells is based on inhibiting Akt-mediated phosphorylation of GLI1 [11]. Furthermore, as Akt was inhibited, caspase 3 and poly (ADP-ribose) polymerase increased in SJ-GBM2, GBM28, and U87MG cells treated with penfluridol, which may indicate that penfluridol enhances apoptosis of GBM cells through Akt suppression. Moreover, the use of GLI1 inhibitors, or knocking down GLI1 by using siRNA or GLI1 CRISPR/Cas9, was studied to determine the role of GLI1 in regulating cancer stem cells through downregulation of OCT4 and Nanog by GLI1 silencing. A low expression level of OCT4 in GLI1 knockout mouse embryonic fibroblasts (MEF) was evident compared to the level of OCT4 in wild-type mice. In vivo, penfluridol showed 65% inhibition based on the volume of GBM tumour, compared controls; the weight of tumours in penfluridol-treated mice was 68% less than that in controls [11]. No side effects were noted regarding the behaviour of mice treated with penfluridol 10 mg/kg/day by oral gavage for 54 days [11].

#### 4.2.3. Induction of Autophagy 

Autophagy is induced by starvation to capture and degrade intracellular proteins and organelles in lysosomes, recycling intracellular components to sustain metabolism and survival [60]. Core autophagy genes (more than 30 genes) are not mutated in cancer [61]. It has been suggested from studies in murine cancer models that autophagy inhibits cancer onset. In contrast, other evidence suggests that autophagy promotes the growth of various advanced cancers, including lung, pancreatic, breast and prostate cancer and melanoma [60]. Dopamine receptors, known as penfluridol targets, are differently involved in autophagy, depending on receptor subtypes. For example, D_2_ and D_3_ receptors are positive regulators and D_1_ and D_5_ receptors are negative regulators [62]. T-type calcium channels, another known target of penfluridol, are also involved in autophagy. The Cav3.1 channel appears to be involved in temozolomide action against GBM through the induction of autophagy [63]. Accordingly, T-type calcium channel blockers also inhibited autophagy and promoted apoptosis in malignant melanoma cells [64]. 

A relationship between autophagy and apoptosis was also observed in pancreatic cells, including Panc-1 cells, AsPC-1 cells, and BxPC-3 cells, treated with penfluridol [12]. This study suggested that autophagy was induced by penfluridol via the upregulation of LC3, a marker of autophagy progression. Moreover, the relationship between autophagy and apoptosis was indicated by decreased penfluridol-induced apoptosis due to blockade of autophagy with inhibitors such as chloroquine, bafilomycinA1 or 3-methyladenine (Figure 3). The role of autophagy and LC3 was also confirmed again by LC3 silencing: a reduced penfluridol effect was noted when LC3B was knocked down by LC3B siRNA before treatment with penfluridol. In addition, formation of pnfluridol-induced autolysosomes was observed through a decreased number of lysosomes, which were fused with autophagosomes during autophagy [12]. In earlier research, penfluridol-treated pancreatic cells also showed an increase in proteins related to cell-cycle arrest such as p21 (cyclin-dependent kinase inhibitor 1A) and cyclin B1 [15], all of which suggests that penfluridol may stimulate apoptosis (Figure 3).

Recently, endoplasmic reticulum (ER) stress was associated with autophagy [65] and tumour suppression [66]. To deal with ER stress, cancer cells use adaptive mechanisms to recover ER proteostasis. This process is called unfolded protein response (UPR), which is regulated by three main stress transducers; inositol requiring enzyme-1α (IRE1α); protein kinase R-like ER kinase (PERK); and activating transcription factor 6 (ATF6) [67,68]. These proteins were upstream down-regulatory factors in several aspects of cancer, such as cell survival, angiogenesis, transformation and resistance to cell death [66,67].

Currently, there is no documented relationship between D_2_ receptors, T-type calcium channels and ER stress in tumourigenesis. However, induction of ER stress was evident in pancreatic cancer cells treated with penfluridol. That is, penfluridol-treated BxPC-3, AsPC-1, and Panc-1 cells experienced increases in ER stress markers in vitro (binding immunoglobulin protein (BIP), CCAAT/enhancer-binding protein [C/EBP] homologous protein [CHOP] and IRE1α). These results were consistent with data from murine models, in which pancreatic tumours were implanted subcutaneously [69]. UPR activation was also documented after penfluridol, which led to nonapoptotic cell death via energy depletion from autophagosome accumulation [32].

In acute myeloid leukemia (AML), penfluridol triggers autophagy. Inhibiting this autophagy increases apoptosis of AML cells, so autophagy induction by penfluridol in AML, via an increase in reactive oxygen species, is cytoprotective [70].

#### 4.2.4. Inhibition of Cholesterol Metabolism

Cholesterol has an important role in cell growth because it is involved in several biological processes and is a key component of cellular membranes. Thus, the maintenance of cholesterol homeostasis is vital to all types of cell, including cancer cells. In previous studies, the role of cholesterol in tumour progression was determined in certain cancers, such as breast and prostate cancer [71,72]. Cholesterol metabolism has become a compelling target for anticancer treatment. 

To date, there is no documented relationship between D_2_-receptor, T-type calcium channels, and cholesterol pathways in tumourigenesis. In 2010, experimental cytotoxicity of six antipsychotic drugs associated with the dysregulation of cholesterol homeostasis was documented. The results showed that antipsychotic drugs selectively inhibit the growth and proliferation of cancer cells compared with normal cells: pimozide had the greater cytotoxic activity [73]. Furthermore, pimozide and olanzapine upregulated important molecules involved in cholesterol homeostasis and also induced some pivotal regulatory genes (namely, *HMGCR*, *LDLR*, and *INSIG1*) involved in cholesterol metabolism [73]. All these genes are established target genes for the sterol regulatory element binding protein (SREBP) transcription factor [74], which plays a vital role in regulating cholesterol synthesis in the livers [75]. 

It has been suggested that pimozide and its derivatives could be used to regulate cholesterol synthesis in the liver. Indeed, penfluridol had a specific downregulatory effect on total cholesterol level in tumours in the B16/F10, LL/2 and 4T1 tumour models, although no statistical difference of serum cholesterol was documented between the penfluridol and control groups. Moreover, penfluridol gradually increased the level of free cholesterol in cells [13]. Overall, these results indicate that penfluridol may specifically dysregulate cholesterol metabolism in cancer cells.

#### 4.2.5. Enhancement of Protein Phosphatase 2A (PP2A) Activity 

PP2A, which comprises a highly conserved group of serine/threonine phosphatases, has an important role in cell transmission pathways. Indeed, *Drosophila* models showed the influential magnitude of PP2A in regulating cell morphology and cell cycles [76]. Moreover, PP2A is involved in regulating autophagy [77] and can therefore be considered an appropriate target for anticancer treatment. Currently, there is no documented relationship between D_2_ receptor, T-type calcium channels, and PP2A in tumourigenesis. However, D_2_-receptor may be associated with PP2A expression in cancer [78].

Induction of PP2A activity might explain the suppressive effect of penfluridol in pancreatic cancer [15]. Moreover, the phosphorylation of two proteins (p70S6K and AKT, which play a key role in cancer) was reduced in penfluridol-treated MIAPaCa-2 cells [15]; p70S6K and AKT were identified as substrates of PP2A [79,80]. Altogether, therefore, PP2A could be considered a potential mechanism for penfluridol action in the treatment of cancer.

#### 4.2.6. Induction of Immunity

Interestingly, dopamine receptor signalling has been linked to anticancer immunity, which has recently entered the spotlight. Inhibition of D_3_ receptor signalling enhances anti-tumour immunity by dendritic cells through increasing antigen cross-presentation for CD8^+^ T-cells [81]. 

MDSCs play a crucial role in the regulation of metastasis and suppression of anti-tumour immunity [82]. These cells infiltrate aggressive GBM [83] and highly express CD11b and Gr1, which could terefore be used as markers for MDSCs [84].

In penfluridol-treated group, spleen weights were increased compared with controls, and this may suggest a correlation between the prevention of malignant tumours and immunity [27]. In addition, decreased CD11b and Gr1 levels in penfluridol-treated MDSCs partly clarified the suppressive effect of penfluridol on GBM cells via the immune system [27]. Furthermore, to elucidate the mechanism by which penfluridol induces anticancer immunity, experiments to measure changes in overproduced proteins in Tregs and macrophages were conducted. FoxP3 and CD4 proteins (markers in Tregs) and CD86 and interleukin-12 (markers in M1 macrophages) decreased in penfluridol-treated groups compared with controls [27]. 

#### 4.2.7. Miscelleneous Mechanisms Involved in Overcoming Resistance

Non-homologous end joining (NHEJ) is the major pathway responsible for repair of ionising radiation (IR)-induced DNA double-strand breaks (DSB) and, accordingly, controls the cellular response to IR [85,86]. NHEJ inhibitors are believed to substantially enhance tumour radiosensitivity and improve the therapeutic efficiency of radiotherapy [85,87]. Penfluridol, an antipsychotic agent, was found in clustered regularly interspaced short palindromic repeats (CRISPR)/Cas9-based screening for NHEJ inhibitors, to increase the amount of broken DNA, as evident from elevated DNA content in comet tails when cells were exposed to 8 Gy of X-rays [85]. Penfluridol also sensitised C6 rat GBM cells to growth inhibition by bleomycin [88].

Paclitaxel is a first choice for patients with triple negative breast cancer (TNBC), but inherited or acquired resistance to paclitaxel results in poor response of these patients [18,89]. The human epidermal growth factor receptor-2 (HER2) and β-catenin pathway is involved in the resistance of TNBC cells to paclitaxel [90]. Interestingly, penfluridol blocks the HER2/β-catenin-signalling pathway in paclitaxel resistant MCF-7 and 4T1 breast cancer cells [19]. Penfluridol also significantly potentiated the tumour growth-inhibitory activity of paclitaxel in an orthotropic breast cancer mouse model [18].

## 5. Needs for Penfluridol Derivatives

**Figure 4:**
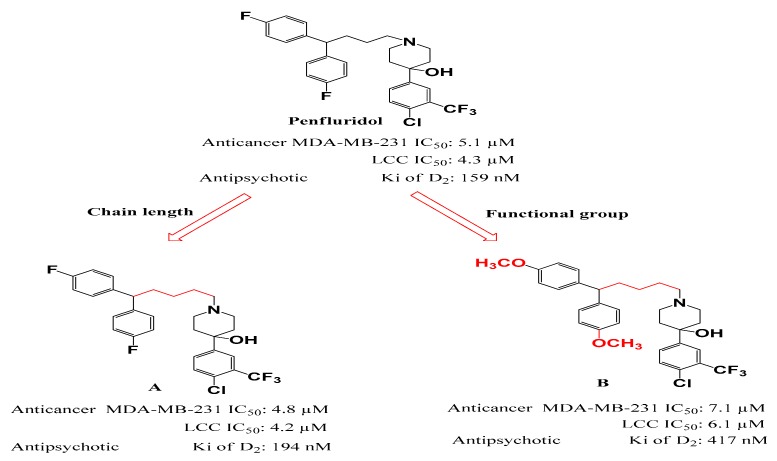
Two synthesized analogs (**A**,**B**) of penfluridol and penfluridol with the anticancer effect against MDA-MB-231 and Lewis Lung Carcinoma (LLC) cell lines and antipsychotic effects Ki of D2 receptor. Modified from Ashraf-Uz-Zaman’s report [9].

The dose used as an anticancer agent is 50 mg/day, which is significantly higher than the dose administered once weekly as an antipsychotic agent [9]. Therefore, neurological side effects such as epilepsy, fatigue, dyskinesias, parkinsonism, akathisia, dystonia and depression, which are observed in antipsychotic drug doses, are more likely to occur when the higher doses associated with anticancer therapy are used. In addition, since penfluridol passes efficently through the BBB, neurological side effects are expected to be greater with the higher doses associated with anticancer therapy. Therefore, in the development of new penfluridol derivatives as anticancer drugs, some of the antipsychotic effects of the compounds were reduced and some of the anticancer effects enhanced (Figure 4). Ashraf-Uz-Zaman et al. [9] first suggested the possibility of deriving penfluridol derivatives with such characteristics.

Regarding the various mechanisms of anticancer activity proposed for penfluridol, no direct target is clear. 

The various anticancer activities of penfluridol cannot be explained by the blockade of dopamine receptors or T-type calcium channels alone. In particular, since novel penfluridol derivatives increase anti-cancer activity and lower neuro-related activity, anti-cancer activity may be due to the blockade of targets other than antipsychotic targets such as dopamine receptors. Research into this possibility is currently underway. In fact, penfluridol was identified as an NHEJ inhibitor with potential applicability as a radiosensitiser [85]. We believe that penfluridol has other targets distinct from dopamine receptors and T-type calcium channels. If a direct target can be identified, then studies of such derivatives of penfluridol may gain further momentum. 

## 6. Perspectives

Recently, as immune-checkpoint inhibitors have shown successful results by regulating the tumour immune environment, it is now recognised that neuronal elements (e.g., activation of sympathetic nerve) are important for cancer development and progression [91,92,93].

In addition, many receptors in nerves are also found in various cancers [94]. So, attempts to treat cancers using neuroactive drugs have become a matter of interest. 

As an antipsychotic, penfluridol is an ‘old’ drug that was approved and used to treat symptoms of schizophrenia via the blockade of dopamine receptors and T-type calcium channels [5,95]. More recently, penfluridol has shown strong anticancer activity in several cancer cell lines. Thus, repurposing penfluridol as a new anticancer drug may be a particularly viable option for anticancer treatment because of major time and cost savings during drug development.

From the treatment perspective, penfluridol has long-lasting efficacy and one dose is effective for up to one week. This long half-life may be advantageous regarding enhanced patients’ compliance and consumption of reduced drug amounts each week. However, anticancer doses of penfluridol might exceed the doses required for antipsychotic use, so combination therapy with other agents plus lower doses of penfluridol might be considered, or new derivatives with stronger anticancer and lower antipsychotic activities may be required. Prerequisites for synthesis of derivatives with these properties are the identification of direct anticancer targets for penfluridol and, if possible, targets other than neuronal GPCRs. Fortunately, recent findings that penfluridol is an NHEJ inhibitor suggest a promise in identifying new targets for the drug [85]. 

To date, the BBB has posed a key challenge to effective anticancer therapy for brain tumours [96]. Penfluridol is a drug taken once a week that passes efficiently through the BBB, which is likely an advantage in the treatment of brain cancer. Penflurdiol attain high concentration in the brain, whereas other anticancer agents may have difficulty crossing the BBB. Penfluridol has anticancer activity in GBM cells (brain cancer cells) [11,27]. Penfluridol also suppresses metastasis of TNBC to the brain and growth of TNBC cells in the brain in vivo [10]. Therefore, the development of novel penfluridol derivatives with the BBB- penetration properties of penfluridol, and with the potential for reduced dosing because of increased anticancer activity, may be helpful for the treatment of brain cancer and cancer metastasis to the brain.

Of course, if penfluridol derivatives were to be used to treat other, non-brain cancers, increased anti-cancer activity with suppression of BBB-penetration properties would be required.

In summary, the presented evidence suggests that penfluridol may have major potential as a treatment for several tumours. This is particularly important as some types of cancer such as GBM, pancreatic and TNBC are widely regarded as being untreatable and have poor survival rates. Recently, in the search for potential agents to manage patients with ‘untreatable cancer’, penfluridol has emerged as a potent anticancer agent in in vitro and in vivo models and with strong suppressive effects against the characteristic features of cancer. If the current limitations of penfluridol as anticancer therapy (e.g., neurological side effects, dose and unknown targets) are overcome, then penfluridol may develop important clinical utility as an anticancer agent. 

## Figures and Tables

**Table 1:** Types of cancer were inhibited by penfluridol.

Types of Cancer	Tested Cell Lines	IC_50_ (µmol/L)	References
Breast cancer	MDA-MB-231, HCC 1806, 4 T1	(5.75–7.5)/24h	[10]
Glioblastoma	GBM 43, GBM 10, GBM 44, GBM 28, GBM 14, T98G, U251 MG, U87MG, SJ-GBM2, CHLA-200	(4.5–10)/24h	[11]
Pancreatic cancer	Panc-1, AsPC-1, BxPC-3	(6.0–6.5)/24h	[12]
Lung Cancer	LCC	4.3/24h	[9]
	LL/2	2.45/48h	[13]
Colon Cancer	CT26	2.74/48h	[13]

## References

[1] Bray F., Ferlay J., Soerjomataram I., Siegel R.L., Torre L.A., Jemal A. (2018). Global cancer statistics 2018: GLOBOCAN estimates of incidence and mortality worldwide for 36 cancers in 185 countries. CA Cancer J. Clin..

[2] Gordon N., Stemmer S.M., Greenberg D., Goldstein D.A. (2018). Trajectories of injectable cancer drug costs after launch in the United States. J. Clin. Oncol..

[3] Howard D.H., Bach P.B., Berndt E.R., Conti R.M. (2015). Pricing in the market for anticancer drugs. J. Econ. Perspect..

[4] Janssen P.A., Niemegeers C.J., Schellekens K.H., Lenaerts F.M., Verbruggen F.J., Van Nueten J.M., Schaper W.K. (1970). The pharmacology of penfluridol (R 16341) a new potent and orally long-acting neuroleptic drug. Eur. J. Pharmacol..

[5] Soares B.G., Lima M.S. (2006). Penfluridol for schizophrenia. Cochrane Database Syst. Rev..

[6] Shintomi K., Yamamura M. (1975). Effects of penfluridol and other drugs on apomorphine-induced stereotyped behavior in monkeys. Eur. J. Pharmacol..

[7] Kline C.L.B., Ralff M.D., Lulla A.R., Wagner J.M., Abbosh P.H., Dicker D.T., Allen J.E., El-Deiry W.S. (2018). Role of Dopamine receptors in the anticancer activity of ONC201. Neoplasia.

[8] Santi C.M., Cayabyab F.S., Sutton K.G., McRory J.E., Mezeyova J., Hamming K.S., Parker D., Stea A., Snutch T.P. (2002). Differential inhibition of T-type calcium channels by neuroleptics. J. Neurosci..

[9] Ashraf-Uz-Zaman M., Sajib M.S., Cucullo L., Mikelis C.M., German N.A. (2018). Analogs of penfluridol as chemotherapeutic agents with reduced central nervous system activity. Bioorg. Med. Chem. Lett..

[10] Ranjan A., Gupta P., Srivastava S.K. (2016). Penfluridol: An antipsychotic agent suppresses metastatic tumor growth in triple-negative breast cancer by inhibiting Integrin signaling axis. Cancer Res..

[11] Ranjan A., Srivastava S.K. (2017). Penfluridol suppresses glioblastoma tumor growth by Akt-mediated inhibition of GLI1. Oncotarget.

[12] Ranjan A., Srivastava S.K. (2016). Penfluridol suppresses pancreatic tumor growth by autophagy-mediated apoptosis. Sci. Rep..

[13] Wu L., Liu Y.Y., Li Z.X., Zhao Q., Wang X., Yu Y., Wang Y.Y., Wang Y.Q., Luo F. (2014). Anti-tumor effects of Penfluridol through dysregulation of Cholesterol homeostasis. Asian Pac. J. Cancer Prev..

[14] Hanahan D., Weinberg R.A. (2011). Hallmarks of cancer: The next generation. Cell.

[15] Chien W., Sun Q.-Y., Lee K.L., Ding L.-W., Wuensche P., Torres-Fernandez L.A., Tan S.Z., Tokatly I., Zaiden N., Poellinger L. (2015). Activation of protein phosphatase 2A tumor suppressor as potential treatment of pancreatic cancer. Mol. Oncol..

[16] Schonthal A.H. (2001). Role of serine/threonine protein phosphatase 2A in cancer. Cancer Lett..

[17] Labi V., Erlacher M. (2015). How cell death shapes cancer. Cell Death Dis..

[18] Gupta N., Gupta P., Srivastava S.K. (2019). Penfluridol overcomes paclitaxel resistance in metastatic breast cancer. Sci. Rep..

[19] Wittekind C., Neid M. (2005). Cancer invasion and metastasis. Oncology.

[20] Viallard C., Larrivee B. (2017). Tumor angiogenesis and vascular normalization: alternative therapeutic targets. Angiogenesis.

[21] Dakir E.H., Pickard A., Srivastava K., McCrudden C.M., Gross S.R., Lloyd S., Zhang S.D., Margariti A., Morgan R., Rudland P.S. (2018). The anti-psychotic drug pimozide is a novel chemotherapeutic for breast cancer. Oncotarget.

[22] Schluter A., Weller P., Kanaan O., Nel I., Heusgen L., Hoing B., Hasskamp P., Zander S., Mandapathil M., Dominas N. (2018). CD31 and VEGF are prognostic biomarkers in early-stage, but not in late-stage, laryngeal squamous cell carcinoma. BMC Cancer.

[23] DeLisser H.M., Christofidou-Solomidou M., Strieter R.M., Burdick M.D., Robinson C.S., Wexler R.S., Kerr J.S., Garlanda C., Merwin J.R., Madri J.A. (1997). Involvement of endothelial PECAM-1/CD31 in angiogenesis. Am. J. Pathol..

[24] Gorgun G.T., Whitehill G., Anderson J.L., Hideshima T., Maguire C., Laubach J., Raje N., Munshi N.C., Richardson P.G., Anderson K.C. (2013). Tumor-promoting immune-suppressive myeloid-derived suppressor cells in the multiple myeloma microenvironment in humans. Blood.

[25] Yang R., Cai Z., Zhang Y., Yutzy W.H., Roby K.F., Roden R.B. (2006). CD80 in immune suppression by mouse ovarian carcinoma-associated Gr-1+CD11b+ myeloid cells. Cancer Res..

[26] Mantovani A., Marchesi F., Malesci A., Laghi L., Allavena P. (2017). Tumour-associated macrophages as treatment targets in oncology. Nat. Rev. Clin. Oncol..

[27] Ranjan A., Wright S., Srivastava S.K. (2017). Immune consequences of penfluridol treatment associated with inhibition of glioblastoma tumor growth. Oncotarget.

[28] Lee C.H. (2018). Epithelial-mesenchymal transition: Initiation by cues from chronic inflammatory tumor microenvironment and termination by anti-inflammatory compounds and specialized pro-resolving lipids. Biochem. Pharmacol..

[29] Alfonso-De Matte M.Y., Moses-Soto H., Kruk P.A. (2002). Calcium-mediated telomerase activity in ovarian epithelial cells. Arch. Biochem. Biophys..

[30] Gutschner T., Diederichs S. (2012). The hallmarks of cancer. RNA Biol..

[31] Yao Y., Dai W. (2014). Genomic instability and cancer. J. Carcinog. Mutagenes..

[32] Hung W.Y., Chang J.H., Cheng Y., Cheng G.Z., Huang H.C., Hsiao M., Chung C.L., Lee W.J., Chien M.H. (2019). Autophagosome accumulation-mediated ATP energy deprivation induced by penfluridol triggers nonapoptotic cell death of lung cancer via activating unfolded protein response. Cell Death Dis..

[33] Weissenrieder J.S., Neighbors J.D., Mailman R.B., Hohl R.J. (2019). Cancer and the Dopamine D2 receptor: A pharmacological perspective. J. Pharmacol. Exp. Ther..

[34] Brami-Cherrier K., Valjent E., Garcia M., Pages C., Hipskind R.A., Caboche J. (2002). Dopamine induces a PI3-kinase-independent activation of Akt in striatal neurons: A new route to cAMP response element-binding protein phosphorylation. J. Neurosci..

[35] Kang S., Dong S.M., Kim B.R., Park M.S., Trink B., Byun H.J., Rho S.B. (2012). Thioridazine induces apoptosis by targeting the PI3K/Akt/mTOR pathway in cervical and endometrial cancer cells. Apoptosis.

[36] Mao M., Yu T., Hu J., Hu L. (2015). Dopamine D2 receptor blocker thioridazine induces cell death in human uterine cervical carcinoma cell line SiHa. J. Obstet. Gynaecol. Res..

[37] Park S.H., Chung Y.M., Ma J., Yang Q., Berek J.S., Hu M.C. (2016). Pharmacological activation of FOXO3 suppresses triple-negative breast cancer in vitro and in vivo. Oncotarget.

[38] Zhou W., Chen M.K., Yu H.T., Zhong Z.H., Cai N., Chen G.Z., Zhang P., Chen J.J. (2016). The antipsychotic drug pimozide inhibits cell growth in prostate cancer through suppression of STAT3 activation. Int. J. Oncol..

[39] Das A., Pushparaj C., Bahi N., Sorolla A., Herreros J., Pamplona R., Vilella R., Matias-Guiu X., Marti R.M., Canti C. (2012). Functional expression of voltage-gated calcium channels in human melanoma. Pigment Cell Melanoma Res..

[40] Antal L., Martin-Caraballo M. (2019). T-type Calcium channels in cancer. Cancers (Basel).

[41] Dziegielewska B., Gray L.S., Dziegielewski J. (2014). T-type calcium channels blockers as new tools in cancer therapies. Pflugers Arch..

[42] Takahashi M., Seagar M.J., Jones J.F., Reber B.F., Catterall W.A. (1987). Subunit structure of dihydropyridine-sensitive calcium channels from skeletal muscle. Proc. Natl. Acad. Sci. USA.

[43] Catterall W.A. (2000). Structure and regulation of voltage-gated Ca^2+^ channels. Annu. Rev. Cell Dev. Biol..

[44] Ertel S.I., Ertel E.A., Clozel J.P. (1997). T-type Ca^2+^ channels and pharmacological blockade: Potential pathophysiological relevance. Cardiovasc. Drugs Ther..

[45] Enyeart J.J., Biagi B.A., Day R.N., Sheu S.S., Maurer R.A. (1990). Blockade of low and high threshold Ca^2+^ channels by diphenylbutylpiperidine antipsychotics linked to inhibition of prolactin gene expression. J. Biol. Chem..

[46] Costello L.C. (2019). The suppression of Prolactin is required for the treatment of advanced Prostate cancer. Oncogen (Westerville).

[47] Valerie N.C., Dziegielewska B., Hosing A.S., Augustin E., Gray L.S., Brautigan D.L., Larner J.M., Dziegielewski J. (2013). Inhibition of T-type calcium channels disrupts Akt signaling and promotes apoptosis in glioblastoma cells. Biochem. Pharmacol..

[48] Kim H., Chong K., Ryu B.-K., Park K.-J., Yu M.O., Lee J., Chung S., Choi S., Park M.-J., Chung Y.-G. (2019). Repurposing Penfluridol in combination with Temozolomide for the treatment of Glioblastoma. Cancers.

[49] Levite M., Chowers Y., Ganor Y., Besser M., Hershkovits R., Cahalon L. (2001). Dopamine interacts directly with its D3 and D2 receptors on normal human T cells, and activates β1 integrin function. Eur. J. Immunol..

[50] Lambert A.W., Ozturk S., Thiagalingam S. (2012). Integrin signaling in mammary epithelial cells and breast cancer. ISRN Oncol..

[51] Muller P.A., Caswell P.T., Doyle B., Iwanicki M.P., Tan E.H., Karim S., Lukashchuk N., Gillespie D.A., Ludwig R.L., Gosselin P. (2009). Mutant p53 drives invasion by promoting integrin recycling. Cell.

[52] Gobira P.H., Ropke J., Aguiar D.C., Crippa J.A., Moreira F.A. (2013). Animal models for predicting the efficacy and side effects of antipsychotic drugs. Braz. J. Psychiatry.

[53] Hedrick E., Li X., Safe S. (2017). Penfluridol represses integrin expression in breast cancer through induction of reactive oxygen species and downregulation of Sp transcription factors. Mol. Cancer Ther..

[54] Safe S., Abdelrahim M. (2005). Sp transcription factor family and its role in cancer. Eur. J. Cancer.

[55] Liou G.-Y., Storz P. (2010). Reactive oxygen species in cancer. Free Radic. Res..

[56] Darnell J.E. (2002). Transcription factors as targets for cancer therapy. Nat. Rev. Cancer.

[57] Clement V., Sanchez P., De Tribolet N., Radovanovic I., Ruiz I., Altaba A. (2007). HEDGEHOG-GLI1 signaling regulates human Glioma growth, cancer stem cell self-renewal, and tumorigenicity. Curr. Biol..

[58] Ignatova T.N., Kukekov V.G., Laywell E.D., Suslov O.N., Vrionis F.D., Steindler D.A. (2002). Human cortical glial tumors contain neural stem-like cells expressing astroglial and neuronal markers in vitro. Glia.

[59] Beaulieu J.-M., Tirotta E., Sotnikova T.D., Masri B., Salahpour A., Gainetdinov R.R., Borrelli E., Caron M.G. (2007). Regulation of Akt signaling by D2 and D3 dopamine receptors in vivo. J. Neurosci..

[60] Amaravadi R., Kimmelman A.C., White E. (2016). Recent insights into the function of autophagy in cancer. Genes Dev..

[61] Ktistakis N.T., Tooze S.A. (2016). Digesting the expanding mechanisms of autophagy. Trends Cell Biol..

[62] Wang D., Ji X., Liu J., Li Z., Zhang X. (2018). Dopamine receptor subtypes differentially regulate autophagy. Int. J. Mol. Sci..

[63] Visa A., Sallán M.C., Maiques O., Alza L., Talavera E., López-Ortega R., Santacana M., Herreros J., Cantí C. (2019). T-type Cav3. 1 channels mediate progression and chemotherapeutic resistance in glioblastoma. Cancer Res..

[64] Das A., Pushparaj C., Herreros J., Nager M., Vilella R., Portero M., Pamplona R., Matias-Guiu X., Martí R.M., Cantí C. (2013). T-type calcium channel blockers inhibit autophagy and promote apoptosis of malignant melanoma cells. Pigment Cell Melanoma Res..

[65] Rashid H.-O., Yadav R.K., Kim H.-R., Chae H.-J. (2015). ER stress: Autophagy induction, inhibition and selection. Autophagy.

[66] Cubillos-Ruiz J.R., Bettigole S.E., Glimcher L.H. (2017). Tumorigenic and immunosuppressive effects of endoplasmic reticulum stress in cancer. Cell.

[67] Hetz C. (2012). The unfolded protein response: controlling cell fate decisions under ER stress and beyond. Nat. Rev. Mol. Cell Biol..

[68] Urra H., Dufey E., Avril T., Chevet E., Hetz C. (2016). Endoplasmic reticulum stress and the hallmarks of cancer. Trends Cancer.

[69] Ranjan A., German N., Mikelis C., Srivenugopal K., Srivastava S.K. (2017). Penfluridol induces endoplasmic reticulum stress leading to autophagy in pancreatic cancer. Tumour Biol..

[70] Wu S.-Y., Wen Y.-C., Ku C.-C., Yang Y.-C., Chow J.-M., Yang S.-F., Lee W.-J., Chien M.-H. (2019). Penfluridol triggers cytoprotective autophagy and cellular apoptosis through ROS induction and activation of the PP2A-modulated MAPK pathway in acute myeloid leukemia with different FLT3 statuses. J. Biomed. Sci..

[71] Freeman M.R., Solomon K.R. (2004). Cholesterol and prostate cancer. J. Cell. Biochem..

[72] Llaverias G., Danilo C., Mercier I., Daumer K., Capozza F., Williams T.M., Sotgia F., Lisanti M.P., Frank P.G. (2011). Role of cholesterol in the development and progression of breast cancer. Am. J. Pathol..

[73] Wiklund E.D., Catts V.S., Catts S.V., Ng T.F., Whitaker N.J., Brown A.J., Lutze-Mann L.H. (2010). Cytotoxic effects of antipsychotic drugs implicate cholesterol homeostasis as a novel chemotherapeutic target. Int. J. Cancer.

[74] Goldstein J.L., DeBose-Boyd R.A., Brown M.S. (2006). Protein sensors for membrane sterols. Cell.

[75] Horton J.D., Goldstein J.L., Brown M.S. (2002). SREBPs: Activators of the complete program of cholesterol and fatty acid synthesis in the liver. J. Clin. Investig..

[76] Janssens V., Goris J. (2001). Protein phosphatase 2A: A highly regulated family of serine/threonine phosphatases implicated in cell growth and signalling. Biochem. J..

[77] Bánréti Á., Lukácsovich T., Csikós G., Erdélyi M., Sass M. (2012). PP2A regulates autophagy in two alternative ways in Drosophila. Autophagy.

[78] Zhang Y., Jiang X., Qin C., Cuevas S., Jose P.A., Armando I. (2015). Dopamine D2 receptors’ effects on renal inflammation are mediated by regulation of PP2A function. Am. J. Physiol.Renal Physiol..

[79] Clerkin J., Naughton R., Quiney C., Cotter T. (2008). Mechanisms of ROS modulated cell survival during carcinogenesis. Cancer Lett.

[80] Wainszelbaum M.J., Liu J., Kong C., Srikanth P., Samovski D., Su X., Stahl P.D. (2012). TBC1D3, a hominoid-specific gene, delays IRS-1 degradation and promotes insulin signaling by modulating p70 S6 kinase activity. PLoS ONE.

[81] Figueroa C., Gálvez-Cancino F., Oyarce C., Contreras F., Prado C., Valeria C., Cruz S., Lladser A., Pacheco R. (2017). Inhibition of dopamine receptor D3 signaling in dendritic cells increases antigen cross-presentation to CD8+ T-cells favoring anti-tumor immunity. J. Neuroimmunol..

[82] Condamine T., Ramachandran I., Youn J.-I., Gabrilovich D.I. (2015). Regulation of tumor metastasis by myeloid-derived suppressor cells. Annu. Rev. Med..

[83] Kohanbash G., Okada H. (2012). Myeloid-derived suppressor cells (MDSCs) in gliomas and glioma-development. Immunol. Investig..

[84] Yang L., Edwards C.M., Mundy G.R. (2010). Gr-1+ CD11b+ myeloid-derived suppressor cells: Formidable partners in tumor metastasis. J. Bone Miner. Res..

[85] Du J., Shang J., Chen F., Zhang Y., Yin N., Xie T., Zhang H., Yu J., Liu F. (2018). A CRISPR/Cas9–based screening for non-homologous end joining inhibitors reveals Ouabain and Penfluridol as Radiosensitizers. Mol. Cancer Ther..

[86] Mahaney B.L., Meek K., Lees-Miller S.P. (2009). Repair of ionizing radiation-induced DNA double-strand breaks by non-homologous end-joining. Biochem J..

[87] Li Y.-H., Wang X., Pan Y., Lee D.-H., Chowdhury D., Kimmelman A.C. (2012). Inhibition of non-homologous end joining repair impairs pancreatic cancer growth and enhances radiation response. PloS ONE.

[88] Hait W., Gesmonde J., Lazo J. (1994). Effect of anti-calmodulin drugs on the growth and sensitivity of C6 rat glioma cells to bleomycin. Anticancer Res..

[89] Hudis C.A., Gianni L. (2011). Triple-negative breast cancer: an unmet medical need. Oncologist.

[90] O’Toole S.A., Beith J.M., Millar E.K., West R., McLean A., Cazet A., Swarbrick A., Oakes S.R. (2013). Therapeutic targets in triple negative breast cancer. J. Clin. Pathol..

[91] Magnon C., Hall S.J., Lin J., Xue X., Gerber L., Freedland S.J., Frenette P.S. (2013). Autonomic nerve development contributes to prostate cancer progression. Science.

[92] Sloan E.K., Priceman S.J., Cox B.F., Yu S., Pimentel M.A., Tangkanangnukul V., Arevalo J.M., Morizono K., Karanikolas B.D., Wu L. (2010). The sympathetic nervous system induces a metastatic switch in primary breast cancer. Cancer Res..

[93] Reiche E.M.V., Nunes S.O.V., Morimoto H.K. (2004). Stress, depression, the immune system, and cancer. Lancet Oncol..

[94] Wang X., Wang Z.-B., Luo C., Mao X.-Y., Li X., Yin J.-Y., Zhang W., Zhou H.-H., Liu Z.-Q. (2019). The prospective value of dopamine receptors on Bio-behavior of tumor. J. Cancer.

[95] Li Y.H., Yu C.Y., Li X.X., Zhang P., Tang J., Yang Q., Fu T., Zhang X., Cui X., Tu G. (2017). Therapeutic target database update 2018: Enriched resource for facilitating bench-to-clinic research of targeted therapeutics. Nucleic Acids Res..

[96] Bhowmik A., Khan R., Ghosh M.K. (2015). Blood brain barrier: A challenge for effectual therapy of brain tumors. Biomed Res. Int..

